# Behavioural change in relation to alcohol exposure in early pregnancy and impact on perinatal outcomes - a prospective cohort study

**DOI:** 10.1186/1471-2393-13-8

**Published:** 2013-01-16

**Authors:** Deirdre J Murphy, Aoife Mullally, Brian J Cleary, Tom Fahey, Joe Barry

**Affiliations:** 1 Academic Department of Obstetrics & Gynaecology, Coombe Women and Infants University Hospital & Trinity College Dublin, Dublin, Dublin 8, Republic of Ireland; 2 School of Pharmacy, Royal College of Surgeons in Ireland, Dublin, Republic of Ireland; 3 HRB Centre for Primary Care Research, Department of Family Medicine and General Practice, Royal College of Surgeons in Ireland, Dublin, Dublin 2, Republic of Ireland; 4 Department of Public Health and Primary Care, Trinity College Dublin, Dublin, Republic of Ireland

**Keywords:** Alcohol exposure, Prospective cohort study, Pregnancy, Perinatal outcomes

## Abstract

**Background:**

There has been limited research addressing whether behavioural change in relation to alcohol exposure in pregnancy results in better perinatal outcomes.

**Methods:**

A cohort study of 6725 women who booked for antenatal care and delivered in a large urban teaching hospital in 2010–2011. A detailed history of alcohol consumption pre-pregnancy and during early pregnancy was recorded at the first antenatal visit with follow-up of the mother and infant until discharge following birth. Adverse perinatal outcomes were compared for ‘non-drinkers’, ‘ex-drinkers’ and ‘current drinkers’.

**Results:**

Of the 6017 (90%) women who reported alcohol consumption prior to pregnancy 3325 (55%) engaged in binge drinking and 266 (4.4%) consumed more than 14 units on average per week. At the time of booking 5649 (94%) women were ex-drinkers and of the 368 women who continued to drink 338 (92%) had a low intake (0–5 units per week), 30 (8%) an excess intake (6-20+ units per week) and 93 (25%) reported at least one episode of binge drinking. Factors associated with continuing to drink in early pregnancy included older maternal age (30–39 years), (OR 1.6; 95% CI 1.3 to 1.8), Irish nationality (OR 3.1; 95% CI 2.2 to 4.3) and smoking (OR 2.6; 95% CI 1.9 to 3.5). Ex-drinkers had similar perinatal outcomes to non-drinkers. Compared to non-drinkers current drinking was associated with an increased risk of intrauterine growth restriction (IUGR) (13% versus 19%, crude OR 1.6; 95% CI 1.1 to 2.2, adjusted OR 1.2; 95% CI 0.8 to 1.8). The greatest risk of IUGR was among women who continued to both drink and smoke, (9% versus 32%, crude OR 4.8; 95% CI 3.3 to 7.0, adjusted OR 4.5; 95% CI 3.1 to 6.7).

**Conclusions:**

Public Health campaigns need to emphasise the potential health gains of abstaining from both alcohol and smoking in pregnancy.

## Background

Many women continue to consume alcohol in pregnancy despite recommendations that they should abstain from alcohol in order to minimise potential risks to the fetus. The advice from the Department of Health in Ireland and the United Kingdom is that alcohol should be avoided during pregnancy. This is similar to the advice by the Surgeon General in the United States that pregnant women or women who may become pregnant should abstain from alcohol. However, the National Institute for Health and Clinical Excellence (NICE) in the United Kingdom recommends that women should be advised to drink no more than one to two units once or twice a week [1]. This conflicting information is likely to be confusing for women who may wish to continue "social" drinking in pregnancy.

A recent Australian study reported that only 41% of pregnant women abstained from alcohol throughout each trimester [2]. An Irish study reported that 81% of women drink alcohol in the peri-conceptional period. However it was not possible to differentiate clearly between pre-pregnancy alcohol use and continuing alcohol use once pregnancy is confirmed [3]. In the Southampton Women’s Survey 70% of women continued to drink in early pregnancy with 10% of drinkers consuming more than four units of alcohol per week [4]. In a Swedish study, 12% of women reported continued alcohol consumption in pregnancy and 5% admitted binge drinking [5]. Despite studies reporting high rates of alcohol use in pregnancy, ascertainment of the true prevalence and extent of alcohol consumption is difficult as under-reporting is common and women's understanding of what constitutes a standard "drink" or "unit" may differ from person to person. Similarly comparisons between different countries can be limited by differences in drinking patterns and different alcohol content of drinks and units [6].

Various studies have associated moderate to heavy alcohol consumption with preterm birth, low birth weight and intrauterine growth restriction (IUGR) [3,7-9]. However, the results are often inconsistent and most studies have shown no significant associations when socio-demographic confounders are taken into consideration. [6] Binge-drinking in pregnancy is also of concern, especially as this pattern of drinking has increased in the female population and is associated with unplanned pregnancy [10,11].

The aim of this study was to use a large cohort of women booking for antenatal care and delivering in a Dublin maternity hospital to investigate the behavioural changes reported in relation to alcohol use in pregnancy and whether there is an impact on adverse perinatal outcomes.

## Methods

A prospective cohort study was carried out using the electronic booking records of women with singleton pregnancies who booked for antenatal care and delivered in a large Dublin maternity hospital between February 2010 and July 2011. The data from the booking interview were linked to the electronic delivery record and neonatal records with information on the infant up until first hospital discharge. Women who booked for antenatal care but delivered elsewhere were not included in the cohort (typically 1-2% of bookings each year).

One of the main objectives of this study was to record with accuracy the prevalence of alcohol consumption in the pre-conceptional period and during early pregnancy. The quality of the booking history was enhanced by use of a carefully structured questionnaire and a laminated drinks chart designed to record alcohol intake and pattern of use with greater accuracy (adapted from Royal College of Psychiatrists guidelines) [12]. This guide to the alcohol content of named alcoholic beverages was made available to all midwives involved in booking patients for antenatal care. All women were asked the following questions at the time of booking (usually between 12 and 14 weeks):

1) Do you drink alcohol?

2) In the three months before this pregnancy how many units of alcohol did you drink each week?

3) In the three months before this pregnancy how many times per month did you drink more than five units of alcohol on a single occasion?

4) How much alcohol do you drink currently?

5) Currently, how many times per month do you drink more than five units of alcohol on a single occasion?

The questions were found to be quick to administer and were acceptable to both patients and staff. Data from the first month was excluded to ensure that all staff had achieved competency with the new questionnaire and in ascertainment of alcohol units consumed. Women who described themselves as complete abstainers from alcohol were described as “non-drinkers”. For women who reported drinking alcohol, the exact number of units consumed per week was recorded. Binge drinking episodes, defined as more than five units of alcohol on a single occasion, were recorded as never, once a month and more than once a month. For the analyses subjects were divided into three groups: “non-drinkers”, “ex-drinkers”, and “current drinkers”. For sub-group comparisons current drinkers consuming 0–5 units per week were termed low alcohol intake. Current drinkers consuming 6–20 + units per week and drinkers reporting any episode of binge drinking during pregnancy were combined into an excess intake/binge category. Further sub-group analyses compared current drinkers who smoked with women who did not drink or smoke.

In addition to alcohol consumption, information on the following maternal characteristics was extracted from the electronic records: maternal age, marital status, socioeconomic group, nationality, public or privately funded antenatal care, parity, planned pregnancy, gestation at booking, smoking, illicit drug use, and referral to a social worker. Maternal age was divided into the following bands: < 20 years, 20–24 years, 25–29 years, 30–34 years, 35–39 years and > 40 years. Socioeconomic groups were classified as professional/manager/employer, home duties, non-manual, manual, unemployed and non-classifiable. Nationality was recorded as either Irish or non-Irish and further sub-divided by region into Western Europe, Asia/Middle East, Eastern Europe, Africa, South America, North America, Australia & New Zealand. Gestational age at booking was divided into < 12 weeks, 12–20 weeks and > 20 weeks. Smokers were defined as women who were current smokers at the time of attendance at their first antenatal visit. Illicit drug users were defined as women who had ever used illicit drugs.

Every woman had an ultrasound scan at the first antenatal visit and a further detailed structural anatomy scan at 20–22 weeks gestation. Gestational age was estimated from the calculation based on first day of the last menstrual period but the booking ultrasound scan estimate was preferred if the dates were uncertain or there was a discrepancy of more than seven days. Perinatal outcome measures included gestational age at delivery, live birth or stillbirth, birth weight, infant gender, infant’s condition at birth including Apgar scores at 1 and 5 minutes, admission to the neonatal unit, any suspected congenital abnormalities and whether resuscitation was required. Detailed data on the neonate were extracted on infants admitted to the neonatal unit including details on ventilation, suspected neonatal abnormalities including Fetal Alcohol Syndrome (FAS) and neonatal death.

Recorded congenital abnormalities were categorised according to the EUROCAT classification system (EUROCAT. *Instructions for the Registration and Surveillance of Congenital Anomalies*. Belfast: European Surveillance of Congenital Anomalies Central Registry, 2009.) Preterm birth was defined as the birth of a live baby at less than 37 weeks gestation. Very preterm birth was defined as the birth of a live baby at less than 32 weeks gestation. Low birth weight was defined as weighing less than 2500 g and very low birth weight as less than 1000 g. Intrauterine growth restriction (IUGR) was defined as a birth weight less than the 10^th^ percentile using individualised birth rate ratios (corrected for maternal height and weight, parity, infant sex, ethnicity and gestation) (http://www.gestation.net). Perinatal deaths included stillbirths or neonatal deaths. Stillbirth was defined as delivery of a baby showing no signs of life at or after 24 weeks gestation. Neonatal death was defined as the death of a baby in the first seven days of life. The perinatal death register, electronic delivery suite and neonatal unit records were used for ascertainment of stillbirths and neonatal deaths.

The analyses were performed using the Statistical Package for Social Sciences (SPSS version 16). Descriptive statistics were used to characterise the study subjects by category of alcohol intake. Comparisons were made between the three groups to identify socio-demographic factors associated with abstaining from alcohol or continuing to consume alcohol in pregnancy. Logistic regression analyses were performed to measure the association between alcohol exposure and adverse perinatal outcomes. The “non-drinker” category was chosen as the comparator for each of the analyses as this was unlikely to be biased by under-reporting and represented a group where we could be certain that there was no alcohol exposure in the peri-conceptional period, even for women with uncertain dates. Further stepwise logistic regression analyses were performed adjusting for potential confounding factors including maternal age, nationality, private health insurance, unplanned pregnancy, smoking, and illicit drug use. These factors were chosen because of their known or possible association with adverse perinatal outcome and because of baseline differences between the groups. Sub-group analyses compared women who consumed alcohol and smoked with women who did not drink or smoke. Results are reported as proportions, crude odds ratios (OR) and adjusted odds ratios (OR) with 95% confidence intervals (CI). All of the chosen variables for the logistic regression models are required data items on the computer system, therefore we did not have missing data as such, and the value "unknown" was rarely used. Binary logistic regression analyses with values coded as "unknown" resulted in a reduced sample size for the particular model but this did not influence the direction or magnitude of any of the associations.

The study received the approval of the Coombe Women and Infants University Hospital’s research ethics committee: Study No. 22–2009. Individual patient consent was not deemed necessary as the study analysed routinely collected data in an anonymised format.

## Results

### Descriptive statistics

A total of 6725 mother-infant pairs were available for analysis. In the three months prior to pregnancy 6017 (90%) women consumed alcohol with 3940 (65%) drinking 0–5 units on average per week, 1799 (30%) drinking 6–14 units per week and 266 (5%) drinking 15-20+ units per week. A total of 3325 (55% of drinkers) reported at least one episode of binge drinking in the previous three months with 1870 of these women (56%) reporting binge drinking more than once a month. At the time of the booking visit 6357 women (95% of drinkers) were ex-drinkers and 368 (5%) were current drinkers. Of the current drinkers 338 (92%) reported drinking no more than 5 units on average per week, 30 (8%) were drinking 6 to 20+ units per week and 93 (25%) reported at least one episode of binge drinking a month. Of the 368 current drinkers 131 (36%) were also smokers.

The characteristics of the women in the cohort in relation to alcohol use in early pregnancy are presented in Table 1. The demographic characteristics of the women were similar to other urban populations in Ireland based on annual maternity statistics. Compared to non-drinkers, ex-drinkers were less likely to be single OR 0.83 (95% CI 0.71 to 0.98), to have an unplanned pregnancy OR 0.70 (95% CI 0.60 to 0.83) or to smoke OR 0.77 (95% CI 0.62 to 0.95) and were more likely to have a professional occupation OR 2.69 (95% CI 2.12 to 3.42) or private obstetric care OR 3.41 (95% CI 2.52 to 4.60). Current drinking was associated with older maternal age (OR 1.64; 95% CI 1.25 to 1.76 and OR 3.27; 95% CI 1.79 to 5.97 for age 30–39 years and > 40 years respectively), Irish Nationality OR 3.05 (95% CI 2.18 to 4.27), smoking OR 2.58 (95% CI 1.90 to 3.49) and a history of illicit drug use OR 2.67 (95% CI 1.80 to 3.95).

**Table 1:** Characteristics of women according to alcohol intake in early pregnancy

**Alcohol intake**	**Non-drinker**	**Ex-drinker**	**Current drinker**	**Odds ratio **^**i**^	**Odds ratio **^**ii**^
**Total**				**95% confidence**	**95% confidence**
**n= 6725**	**n= 708 (%)**	**n= 5649 (%)**	**n= 368 (%)**	**intervals**	**intervals**
Maternal age					
< 20 years	27 (3.8)	161 (2.8)	10 (2.7)	0.99 (0.65-1.51)	0.99 (0.46-2.10)
20-29 years ^∫^	312 (44.0)	1884 (33.3)	117 (31.8)	1.00	1.00
30-39 years	347 (49.0)	3362 (59.4)	214 (58.1)	1.60 (1.36-1.89)*	1.64 (1.25-1.76)*
> 40 years	22 (3.1)	252 (4.5)	27 (7.3)	1.90 (1.21-2.98)*	3.27 (1.79-5.97)*
Single Marital status	302 (42.7)	2211 (39.1)	179 (48.6)	0.83 (0.71-0.98)*	1.24 (0.97-1.61)
Socioeconomic group					
Professional	149 (21.0)	1823 (32.3)	101 (27.4)	2.69 (2.12-3.42)*	1.24 (0.86-1.78)
Home duties ^∫^	159 (22.5)	723 (12.8)	87 (23.6)	1.00	1.00
Non-manual	241 (34.0)	2275 (40.2)	111 (30.2)	2.08 (1.67-2.58)*	0.84 (0.60-1.19)
Manual	35 (4.9)	219 (3.9)	13 (3.5)	1.38 (0.93-2.04)	0.68 (0.34-1.35)
Unemployed	78 (11.0)	384 (6.8)	41 (11.1)	1.08 (0.80-1.46)	0.96 (0.61-1.52)
Non-classifiable	46 (6.5)	229 (4.0)	15 (4.1)	1.09 (0.76-1.57)	0.60 (0.32-1.13)
Irish Nationality	472 (66.7)	4732 (83.6)	317 (86.1)	2.56 (2.15-3.04)*	3.05 (2.18-4.27)*
Private Health Care	48 (6.8)	1137 (20.1)	35 (9.5)	3.41 (2.52-4.60)*	1.42 (0.90-2.24)
Nulliparous	270 (38.1)	2548 (45.0)	126 (34.2)	1.31 (1.12-1.54)*	0.84 (0.64-1.09)
Unplanned pregnancy	268 (37.9)	1684 (29.8)	147 (39.9)	0.70 (0.60-0.83)*	1.10 (0.86-1.44)
Gestation at booking					
< 12 weeks ^∫^	254 (35.9)	2299 (40.6)	118 (32.1)	1.00	1.00
12-20 weeks	400 (56.5)	3078 (54.4)	215 (58.4)	0.85 (0.72-1.00)	1.16 (0.88-1.52)
> 20 weeks	54 (7.6)	264 (4.7)	33 (9.0)	0.54 (0.39-0.74)*	1.32 (0.81-2.14)
Smoker	127 (17.9)	808 (14.3)	131 (35.6)	0.77 (0.62-0.95)*	2.58 (1.90-3.49)*
Illicit drug use (ever)	52 (7.3)	476 (8.4)	64 (17.4)	1.17 (0.86-1.57)	2.67 (1.80-3.95)*
Social worker referral	32 (4.5)	105 (1.9)	15 (4.1)	0.41 (0.27-0.61)*	0.88 (0.47-1.66)

^i^ Ex-drinkers versus Non-drinkers.

^ii^ Current drinkers versus Non-drinkers.

^∫^ Reference category.

* p<0.05.

### Perinatal outcomes in relation to alcohol exposure

Ex-drinkers had very similar perinatal outcomes to non-drinkers (Table 2). Compared to non-drinkers, current drinking was associated with an increased risk of intrauterine growth restriction (IUGR) (12.9% versus 18.8%, crude OR 1.56 (95% CI 1.11 to 2.20). The association was attenuated on controlling for potential confounding factors, adjusted OR 1.23 (0.83 to 1.83). Current drinkers who had a low intake of alcohol (1–5 units) had similar or better perinatal outcomes than non-drinkers (Table 3). Compared to non-drinkers, women who consumed excess alcohol (> 5 units) or who reported any episode of binge drinking in pregnancy had an increased risk of IUGR (12.9% versus 26.5%, crude OR 2.41; 95% CI 1.47 to 3.98, adjusted OR 1.43; 95% CI 0.83 to 2.49). Smoking, extremes of maternal age, and non-Irish country of origin caused the greatest attenuation in risk estimates after adjustment in the logistic regression models.

**Table 2:** Perinatal outcomes according to reported alcohol behaviour at first antenatal visit in pregnancy

**Alcohol intake**	**Non-drinker**	**Ex-drinker**	**Current drinker**	**Ex-drinker versus Non-drinker**	**Current drinker versus Non-drinker**
	**n= 708 (%)**	**n= 5649 (%)**	**n= 368 (%)**	**Crude OR (95% CI)**	**Crude OR (95% CI)**
				**Adjusted **^**i **^**OR (95% CI)**	**Adjusted **^**i **^**OR (95% CI)**
Preterm birth < 37 weeks	53 (7.5)	345 (6.1)	23 (6.2)	0.79 (0.59-1.07)	0.82 (0.49-1.35)
				1.11 (0.75-1.63)	0.56 (0.32-1.12)
Very preterm birth < 32 weeks	11 (1.6)	71 (1.3)	4 (1.1)	0.79 (0.42-1.51)	0.69 (0.22-2.18)
				0.87 (0.41-1.84)	0.47 (0.14-1.16)
Low birth weight < 2500g	41 (5.8)	259 (4.6)	22 (6.0)	0.77 (0.55-1.08)	1.02 (0.60-1.74)
				0.82 (0.55-1.21)	0.69 (0.39-1.22)
Very low birth weight < 1000g	5 (0.7)	24 (0.4)	3 (0.8)	0.59 (0.23-1.55)	1.14 (0.27-4.79)
				0.54 (0.20-1.46)	0.91 (0.20-4.13)
Intrauterine growth restriction ^ii^	91 (12.9)	640 (11.3)	69 (18.8)	0.87 (0.68-1.10)	1.56 (1.11-2.20)*
				0.90 (0.68-1.18)	1.23 (0.83-1.83)
Apgar score <3 at 1 minute	8 (1.1)	55 (1.0)	3 (0.8)	0.85 (0.40-1.79)	0.71 (0.19-2.70)
				1.12 (0.44-2.89)	0.56 (0.14-2.25)
Apgar score <7 at 5 minutes	6 (0.8)	45 (0.8)	4 (1.1)	0.92 (0.39-2.18)	1.28 (0.36-4.55)
				1.12 (0.39-3.22)	0.68 (0.18-2.63)
Admitted to neonatal unit	43 (6.2)	271 (4.8)	20 (5.4)	0.77 (0.55-1.07)	0.92 (0.53-1.60)
				0.76 (0.51-1.13)	0.63 (0.35-1.13)
Congenital abnormality (any)	15 (2.1)	46 (0.8)	5 (1.4)	0.37 (0.21-0.81)*	0.63 (0.23-1.74)
				0.40 (0.21-0.77)*	0.73 (0.25-2.12)
Perinatal death	5 (0.7)	20 (0.4)	2 (0.5)	0.62 (0.21-1.81)	0.76 (0.15-3.92)
				0.87 (0.25-3.02)	0.80 (0.14-4.53)

^i^ Adjusted for maternal age, Irish nationality, unplanned pregnancy, private healthcare, smoker, illicit drug use.

^ii^ Customised birth weight < 10^th^ percentile.

* p<0.05.

**Table 3:** Perinatal outcomes according to units of alcohol intake reported at the first antenatal visit in pregnancy

**Alcohol intake**	**Non-drinker**	**Low intake**	**Excess intake / binge**	**Low intake versus Non-drinker**	**Excess intake / binge versus Non-drinker**
	**(0 units)**	**(1–5 units)**	**(>5 units)**	**Crude OR 9(5% CI)**	**Crude OR (95% CI)**
	**n= 708 (%)**	**n= 270 (%)**	**n= 99 (%)**	**Adjusted **^**i **^**OR (95% CI)**	**Adjusted **^**i **^**OR (95% CI)**
Preterm birth < 37 weeks	53 (7.5)	14 (5.2)	10 (10.2)	0.67 (0.36-1.23)	1.62 (0.76-3.45)
				0.51 (0.27-0.96)*	0.83 (0.37-1.82)
Very preterm birth < 32 weeks	11 (1.6)	1 (0.4)	3 (3.0)	0.23 (0.03-1.81)	1.99 (0.55-7.28)
				0.17 (0.02-1.35)	1.35 (0.33-5.47)
Low birth weight < 2500g	41 (5.8)	13 (4.8)	9 (9.2)	0.81 (0.43-1.54)	1.62 (0.76-3.45)
				0.60 (0.31-1.18)	1.00 (0.44-2.26)
Very low birth weight < 1000g	5 (0.7)	1 (0.4)	2 (2.0)	0.52 (0.06-4.43)	2.89 (0.55-15.09)
				0.40 (0.04-3.70)	2.50 (0.41-15.11)
Intrauterine growth restriction ^ii^	91 (12.9)	42 (15.6)	26 (26.5)	1.25 (0.84-1.86)	2.41 (1.47-3.98)*
				1.04 (0.68-1.59)	1.43 (0.83-2.49)
Apgar score <3 at 1 minute	8 (1.1)	1 (0.4)	2 (2.0)	0.32 (0.04-2.60)	1.80 (0.38-8.59)
				0.30 (0.04-2.50)	1.27 (0.24-6.78)
Apgar score <7 at 5 minutes	6 (0.8)	3 (1.1)	4 (4.0)	1.10 (0.18-2.67)	4.94 (1.37-17.83)*
				1.09 (0.11-2.73)	2.34 (0.55-9.90)
Admitted to neonatal unit	43 (6.2)	10 (3.7)	10 (10.2)	0.62 (0.30-1.25)	1.82 (0.88-3.76)
				0.47 (0.22-0.97)*	1.30 (0.59-2.85)
Congenital abnormality (any)	15 (2.1)	3 (1.1)	2 (2.0)	0.51 (0.15-1.78)	0.95 (0.21-4.21)
				0.63 (0.18-2.28)	1.08 (0.23-5.13)
Perinatal death	5 (0.7)	1 (0.4)	1 (1.0)	0.52 (0.06-4.43)	1.43 (0.17-12.36)
				0.61 (0.07-5.61)	1.28 (0.13-12.64)

^i^ Adjusted for maternal age, Irish nationality, unplanned pregnancy, private healthcare, smoker, illicit drug use.

^ii^ Customised birth weight < 10^th^ percentile.

* p<0.05.

### Sub-group analyses of women who drink and smoke

The risk of IUGR was greatly increased among women who continued to both drink and smoke (9.1% of non-drinker non-smokers versus 32.1% of drinkers and smokers, crude OR 4.78; 95% CI 3.26 to 7.01, adjusted OR 4.52; 95% CI 3.08 to 6.68). The risk of IUGR was increased further in women who reported excess alcohol or binge drinking and smoked (9.1% versus 37.7%, crude OR 5.98; 95% CI 3.39 to 10.57, adjusted OR 5.60; 95% CI 3.16 to 9.92).

## Discussion

### Summary of main findings

This study found that a large number of women attending for antenatal care made a decision to abstain from alcohol by the time of the booking visit and that these women have perinatal outcomes similar to non-drinkers. Continued alcohol consumption in pregnancy is associated with older maternal age and Irish Nationality although the majority of these women have a low alcohol intake. Of those women who continue to drink in pregnancy, a significant minority drink excess alcohol or binge drink. There is a strong association between alcohol use in pregnancy and smoking and these factors combined have the most profound effect on fetal growth, with a four-fold increased risk of intrauterine growth restriction.

### Strengths and limitations of the study

The population consisted of a complete geographical cohort of women attending a large urban maternity hospital over a one year period. The data were collected prospectively by qualified midwives using a standardised computer guided interview and a systematic approach to ascertaining information on the pattern and amount of alcohol consumed prior to pregnancy and at the time of the booking visit. Nonetheless the data on alcohol intake relied on self-reporting by the pregnant woman at one time point and it is possible that the amount of alcohol consumed is under-reported. It is unlikely that alcohol intake is over-estimated. As the data were collected at the first antenatal visit the potential for recall bias was limited. We did not have data on alcohol intake later in pregnancy and it is possible that maternal alcohol intake alters as pregnancy progresses. Neonates were examined prior to first hospital discharge and longer term follow-up would be required to detect and confirm cases of fetal alcohol syndrome (FAS), fetal alcohol spectrum disorder and neuro-developmental deficit. Of note, ex-drinkers had many positive health characteristics in comparison to non-drinkers (who might be considered the healthiest group), therefore comparative analyses need to account for potential confounding factors working in opposite directions.

### Comparison with existing literature

In our previous retrospective cohort study women were asked how many units of alcohol they consumed each week prior to becoming pregnant [3]. No explanation of a “unit” of alcohol or the alcohol content of different alcoholic drinks was provided and binge drinking was not specifically addressed. Kesmodel *et al.* have suggested that, in studies focusing on the prevalence of alcohol intake among pregnant women, questions on intake should focus on a time period of at least two weeks [13]. Examining alcohol intake over a more prolonged period of time is preferable when exploring the effect of alcohol on pregnancy outcomes which are likely to be caused by sustained exposure to alcohol, such as low birth-weight. The likelihood of congenital malformations is more likely to be influenced by peak blood alcohol concentration such as that achieved by binge-drinking [11]. These issues were addressed at the antenatal booking interview in order to improve the reporting of total alcohol intake, timing of consumption and patterns of drinking for this cohort of women.

Understanding women’s attitudes and behaviour is essential in order to design and implement effective health promotion strategies for pregnancy. Similar to our study, a Danish study reported at least one episode of binge-drinking among 50% of women during the time from their last menstrual period until pregnancy recognition [11,13]. An Australian cross-sectional survey of women aged 18 to 45 years reported that women’s past pregnancy and current drinking behaviour were the strongest predictors of intention to consume alcohol in a future pregnancy [14]. In a US telephone interview of control women from the National Birth Defects Prevention Study, pre-pregnancy binge drinking was a strong predictor of both drinking during pregnancy and binge drinking during pregnancy [15]. The highest prevalence of alcohol consumption in a US study from Maryland was reported by mothers who were non-Hispanic white, aged 35 years or older and college graduates [16]. Counselling about the risks of alcohol in pregnancy was least prevalent among the groups of women with the highest rates for drinking. Several studies confirmed the association we found between alcohol consumption in pregnancy and cigarette smoking [14,15,17].

Henderson et al. performed a systematic review of the effects of low to moderate prenatal alcohol exposure on pregnancy outcome [6]. In keeping with our study they found no consistent evidence of adverse perinatal effects at low levels of consumption. In support of this finding, a Swedish study demonstrated that low to moderate alcohol consumption, as measured by questionnaire and blood biomarkers, was not associated with negative newborn outcomes [5]. However, a Danish study reported an association between moderate alcohol intake during pregnancy and an increased risk of fetal death in the first and early second trimester [18]. As with our study there was no increased risk of fetal death after 16 weeks gestation. A cohort study in Australia investigated the effect of maternal alcohol intake on fetal growth and preterm birth [2]. The investigators collected data for the three-month period pre-pregnancy and for each trimester separately. However, the data were collected postpartum and relied on recall. In keeping with our study, they found that the rate of low birth weight infants was highest among those exposed to either binge or heavy drinking in pregnancy. In a Dutch prospective cohort study of light and moderate maternal alcohol consumption, cross-sectional analyses in mid- and late pregnancy showed no consistent associations between the number of alcoholic drinks consumed and fetal growth characteristics [19]. However a meta-analysis of the dose–response relationship between alcohol consumption before and during pregnancy on perinatal outcomes indicates that heavy alcohol consumption increases the risks of low birthweight, preterm birth and small for gestational age (SGA) [9].

### Implications for practice

Current government policies in Ireland, the United Kingdom and the United States of America recommend that it is in a child's best interests for women not to consume any alcohol during pregnancy [12]. In contrast, the NICE guidelines in the United Kingdom recommend that women should be advised to drink no more than one to two units once or twice a week [1]. It is clear from this study that large numbers of women are drinking in the peri-conceptional period, some at high levels. Although the majority of women report abstaining from alcohol at the time of the booking visit there is still a significant minority who continue to binge drink or drink to excess. There is a need for more accurate ascertainment of alcohol consumption so that high risk drinkers can be identified early in pregnancy and offered appropriate interventions to reduce the risk to the developing fetus. The prevention of high-risk drinking in pregnancy could reduce adverse perinatal outcomes with implications for the affected families and cost to the healthcare system. Public health campaigns need to address the high rate of binge drinking among women and the association with unplanned pregnancy and late booking for antenatal care. The combined risks of alcohol and smoking highlights the importance of addressing all types of unhealthy behaviour in pregnancy and the pre-pregnancy period.

## Conclusions

This study emphasises the need for improved detection and management of problem alcohol use in pregnancy and for early intervention in order to minimise the risks to the developing fetus. Public Health campaigns need to emphasise the potential health gains of abstaining from both alcohol and smoking in pregnancy.

## Competing interests

The authors declare that they have no competing interests.

## Authors’ contributions

DJM (guarantor) had the original idea for the study and, with all co-authors carried out the design. DJM and JB obtained funding. DJM, AM and BC were responsible for data cleaning. DJM carried out the analyses. DJM and AM drafted the manuscript which was revised by all authors. All authors read and approved the final manuscript.

## Pre-publication history

The pre-publication history for this paper can be accessed here:

http://www.biomedcentral.com/1471-2393/13/8/prepub
